# Dynamically Reconfigurable Encryption and Decryption System Design for the Internet of Things Information Security

**DOI:** 10.3390/s19010143

**Published:** 2019-01-03

**Authors:** Zhu Wang, Yan Yao, Xiaojun Tong, Qinghua Luo, Xiangyu Chen

**Affiliations:** 1 Department of Information Science and Engineering, Harbin Institute of Technology-Weihai, No.2 Wenhuaxilu, Huancui District, Weihai 264200, China; luoqinghua081519@163.com (Q.L.); Hitcxy1994@163.com (X.C.); 2 Department of Computer Science and Technology, Harbin Institute of Technology-Weihai, No.2 Wenhuaxilu, Huancui District, Weihai 264200, China; tong_xiaojun@163.com

**Keywords:** information security, Internet of Things, encryption and decryption system, FPGA, resources consumption, cyber-security

## Abstract

Information security is the foundation for building trust between the Internet of Things (IoT) and its users. Due to the sharp increase of information quantity and the limitation of hardware resources, it is difficult to maintain the high performance of hardware equipment, while also enhancing information security. To solve the problem of high consumption and low flexibility of multiple cryptographic algorithms hardware implementation, we have designed the Dynamically Reconfigurable Encryption and Decryption System, which is based on Field Programmable Gate Array. Considering the functional requirements, the cryptographic algorithm reconfigurable module files stored in External Memory could be configured dynamically into the assigned on-chip Reconfigurable Partition, supported by Core Controller and the Reconfiguration Control Platform. The experiment results show that, compared with the Static Encryption and Decryption System, our design reduces the logic resources by more than 30% and completes the algorithm swapping at the configuration speed of 15,759.51 Bytes/ms. It indicates that our design could reduce logic resources consumption and improve utilization efficiency and system flexibility.

## 1. Introduction

In recent years, the rapid development of the Internet and communication technology has resulted in intelligent devices penetrating every aspect of human life [1]. With the advantages of high speed computer network processing and wireless data communication, the Internet of Things (IoT) realizes the interconnection of objects distributed in different locations and deepens the popularity and progress of the information age. At present, IoT has been widely used in industrial monitoring, urban management, smart home, and intelligent transportation [2,3,4]. Furthermore, it is estimated that by 2020 there will be 2–5 billion devices connected through IoT [5,6]. In order to prevent information leakage in complex and changeable application environments, protecting information security has become a hot topic for researchers and technical companies.

In the process of IoT data transmission, cryptographic algorithms are the most commonly effective means to guarantee data security [7,8,9]. The data volumes expansion and the IoT commoditization give computer hardware higher requirements for the speed, power consumption, and cost. In addition, to enhance the cryptographic algorithms cracking difficulty, the complexity of the algorithms undoubtedly increases the burden for the limited resource hardware implementation. Compared with the Application Specific Integrated Circuit (ASIC) and the digital signal processor (DSP), Field Programmable Gate Array (FPGA), a semi-custom circuit in the ASIC field, not only reduces the development cycle of custom circuit, but also overcomes the shortcomings of the original programmable device limited gates. It has the advantages of fast processing speed, rich functions, high flexibility, and ultra-low power consumption. In recent years, FPGA has become an ideal platform for IoT to cope with big data and cloud computing [9].

Data encryption in IoT mainly includes information encryption and message authentication, which protects information security and detects information tampering [10]. Different cryptographic algorithms meet different encryption requirements due to their unique characteristics. Therefore, the encryption and decryption system needs to reserve a variety of algorithms to facilitate consumers’ flexible uses. However, the encryption and decryption system building on the FPGA faces three problems: First, the implementation of multiple algorithms will further increase the hardware logic resources consumption, which may exceed the total amount of on-chip resources. Second, only one algorithm is used to process data at the same time, while other algorithms still occupy a large amount of logic resources, which reduces resources utilization. Third, when modifying and updating the cryptographic algorithm, the system must stop all work processes and perform downtime maintenance, which limits flexibility.

In order to enhance information security and hardware implementation performance of IoT, the encryption and decryption system based on dynamically reconfigurable technology provides a solution. In our previous work [11], a dynamic reconfigurable control platform supporting multiple cryptographic algorithms was built, wherein the host computer user can flexibly select the cryptographic algorithms and the dynamic switching was implemented in the allocated area without all cryptographic algorithms occupying the hardware resources. Therefore [11], this paper further optimizes the hardware implementation and completes the design of Dynamic Reconfigurable Encryption and Decryption System (DREDS). Moreover, this paper optimizes the implementation of AES, 3DES algorithm, and the Static Encryption and Decryption System (SEDS), as well as enriches the data of hardware resources utilization. Section 2 introduces the research status of cryptographic algorithms in IoT and the application of dynamically reconfiguration in recent years. Section 3 displays the algorithm principle of Advanced Encryption Standard (AES) and Triple Data Encryption Standard (3DES) and proposes algorithms optimization schemes. Section 4 is the main content of the design. It introduces the overall structure of DREDS and the design process of Reconfiguration Control Platform and Core Controller. After the DREDS was tested on Vivado2016.4 software, Section 5 shows the hardware implementation and data validation of the proposed system on the Xilinx ZYNQ-7000 series of FPGA, and analyzes the performance of the proposed system and the SEDS. It is worth mentioning that we proposed the speed index of algorithms switching compared with similar papers.

## 2. Related Work

In recent years, many researchers have conducted studies on cryptographic algorithms in the field of IoT. Cheema and Julka [8] proposed a dynamic password access mechanism to store the same key matrix on all nodes. The sender did not need to send a simple key but the storage coordinates of the key matrix and the receiver obtained the key from the matrix according to the coordinates, which effectively improves the security of key transmission. Pandey et al. [9] proposed a Very Large Scale Integration (VLSI) architecture, with high performance and a limited area of 64-bit data path for lightweight cipher PRESENT, which realized high throughput rate and low power consumption on FPGA. For meeting the security requirements of different application environments, different algorithms are required for the encryption and decryption system to process data. Banerjee et al. [12] designed a tunable encryption system and the users had freedom to select the preferred encryption type from multiple types listed in the Kerberos configuration file, improving system flexibility. Jasim and Imad [13] proposed a hybrid Short Message Services (SMS) protocol design with AES and Rivest Cipher 4 (RC4) algorithms, to ensure the smart house security. In this design, the confidential and random secure communication in IoT environment was provided, but there were still a certain amount of overhead both in time and space. Hossain et al. [14] implemented a reconfigurable encryption system on the FPGA. The users could select one of AES, Data Encryption Standard (DES), and 3DES algorithms to encrypt by their requirements. In this design, the system flexibility was improved, but the logic resources were wasted. To balance the relevance between system flexibility and hardware resource consumption, the emergence of dynamically reconfigurable technology provided a better solution.

Dynamically reconfigurable technology was first widely used in the aerospace field to solve the problem of functional reconstruction after device damage. It could relay out the existing circuit to restore the original function. Therefore, it has two significant features: First, the logic function can be switched using time division multiplexing without system power-off, which greatly improves the system flexibility and reduces the delay of switching function. Second, dynamically realizing functions switching on limited logic could reduce the resources occupation besides ensuring flexibility [15]. With the development of the electronic information industry, dynamically reconfigurable technology has gradually penetrated into industrial automation [16,17], wireless network [17,18,19], Information security [20,21], and other fields. Niar et al. [22] proposed a dynamically reconfigurable multilevel infrastructure for intelligent and efficient traffic management in Emergency and Disaster Management (EDM). High performance computing and communication bandwidth were realized using embedded multicore architectures of MPSoc and FPGA-based dynamically reconfigurable embedded systems. Ben et al. [23] presented a dynamically reconfigurable architecture for rendering low complex 3D objects. It was an adaptive elementary Graphics Processing Unit (GPU) that used the Microblaze Central Processing Unit (CPU) with a variable set of coprocessors. A controller was used to manage the internal architecture of the GPU to meet different application requirements. Silva et al. [24] presented a new dynamically reconfigurable ΣΔ-based transmitter architecture supporting multichannel multimode data transmission, for providing a highly flexible data transmission scheme for the application of cognitive radio. In order to highlight the contribution of our proposed system, the different between the DREDS and the related works is compared in Table 1.

## 3. AES and Data Encryption Standard (3DES) Implementation

In order to make the cryptographic algorithms more conducive to hardware implementation, we have optimized the sub-steps of AES and 3DES in terms of resources saving and processing speed improving.

### 3.1. 3DES Optimizaiton

**Figure 1:**
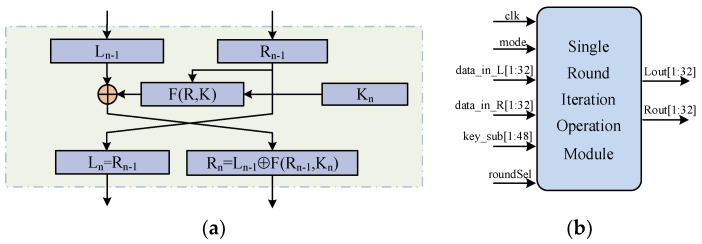
The details of the data encryption standard(DES) and 3DES single iteration operation: (**a**) The single iteration step of DES. (**b**) The single iteration implementation of 3DES based on logical multiplexing.

3DES is a block cipher developed from DES. It uses two different keys to encrypt the data three times to provide higher security guarantee [25]. Both the data block and the key length of DES is 64 bits. The DES encryption process is performed as follows: First, the plaintext is transformed to 64 bits muddled data through Initial Permutation and the data is divided into two parts, L_0_ and R_0_. Then, 16 iteration steps are performed, as shown in Figure 1a. At the same time, the initial key is subjected to 16 rounds of expansion and the subkey K_n_ is obtained to participate in each encryption iteration. After the iterations are completed, the L_15_ and R_15_ are interchanged again. The results of R_16_ and L_16_ are spliced into 64 bits and the Inverse Initial Permutation is performed to obtain the final ciphertext output [26]. The DES decryption direction is the reverse of the encryption process, using subkeys in reverse order.

3DES involves implementing DES three times, which requires 48 iterations. If each iteration is serially designed in the circuit, a large amount of logic resources will be consumed. Based on the design idea of logical multiplexing, we designed the single round iteration operation as a circuit module, as shown in Figure 1b. Through the control of the clock signal and the counter, the module is cyclically called to complete the multiple rounds of data iteration. In order to reduce resources consumption and processing time as much as possible without changing the function, we optimized the logic implementation of subkey generation and the F(R,K) function.

#### 3.1.1. Subkey Generation

According to the three processing steps of the subkey generation—i.e., the permutation and compression of PC-1, the 16 rounds cyclic shift, and the permutation and compression of PC-2—it can be seen that there is a unique correspondence between the subkey and the initial key. Therefore, the relationship between the subkey and the initial key could be calculated in advance and flow into each round of encryption operation by direct assignment. This optimization method only consumes a small amount of hardware resources. With the input of the initial key, each subkey is expanded in one clock cycle, which effectively reduces the time consumption of the calculation process.

#### 3.1.2. F(R,K) Function

The F(R,K) function mainly includes extension permutation, XOR operation, substitution transformation, and P table permutation operation. In order to simplify the operation and make it suitable for the working characteristics of FPGA, we optimize each step of the F(R,K) function. The extension permutation and P table permutation operation belong to the position substitution operation, so the data of the position in the permutation table can be connected to the corresponding linear variables by means of the assignment and concatenation, avoiding the consumption of the register resources and improves the processing speed. The XOR operation is realized by the multiple XOR gates formed by Look-Up-Table (LUT). The data is input into the XOR gate through the input port, without the clock signal control. When the input data is changed, the output data is directly obtained. The substitution transformation is a key part of ensuring algorithm security. Without register variables, we directly used the input data as a sensitive signal for detection. When the input data changes, the corresponding data in LUT is immediately updated to the linear variable and performs subsequent operations, reducing unnecessary clock cycles and speeding up the data processing process.

### 3.2. AES Optimization

The AES is a symmetric block cipher with 128-bit data block as input [27]. The 128-bit, 192-bit, and the 256-bit keys are selectable, corresponding to 10, 12, and 14 rounds as round transformation. The SubBytes, ShiftRows, MixColumns, and AddRoundKey are the four steps in a normal round. The AES algorithm operations are performed in the State, represented by a 4 × 4 bytes matrix.

**Figure 2:**
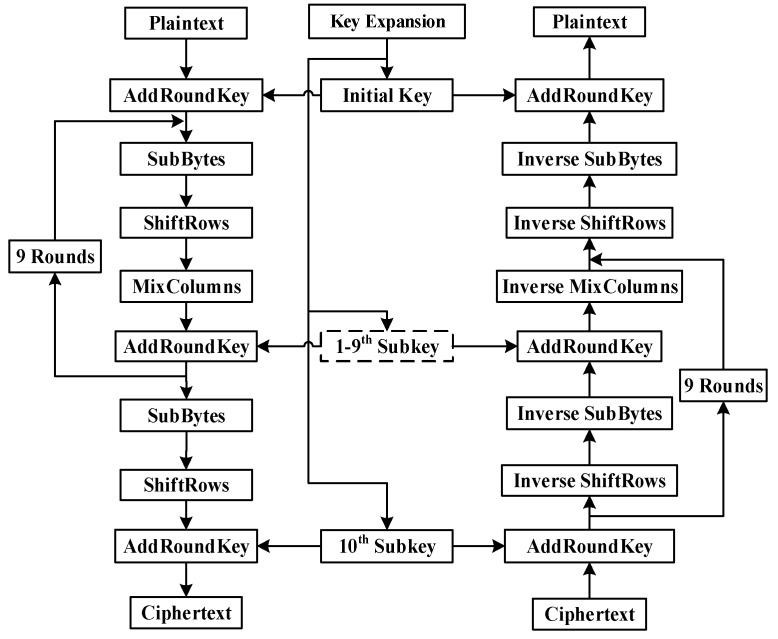
The data flow diagram of Advanced Encryption Standard (AES)-128 encryption and decryption implementation.

In this paper, the data encryption and decryption was conducted using AES with a 128-bit key. The data flow diagram of AES-128 encryption and decryption is shown in Figure 2. For high performance of FPGA implementation, the complicated processing of SubBytes/Inverse SubBytes and MixColumns/Inverse MixColumns are optimized.

#### 3.2.1. SubBytes/Inverse SubBytes

The multiplication inverse operation and affine transform of SubBytes are complicated. Until now, the commonly used implementation method is to calculate the substitution result of all numbers of GF(2^8^) in advance and store it in a 16 × 16 Bytes S-box [28]. In the SubBytes, each byte in the State uses an S-box, so a total of 16 S-boxes are required. At this point, the memory resources on the FPGA become a better implementation. This article uses an S-box to be stored in Random Access Memory (RAM) and retrieves internal data when needed. Xilinx’s FPGA has two kinds of memory resources: Distributed RAM and Block RAM (BRAM). Distributed RAM is formed by the combinational logic resources in SLICEM, which still consumes a lot of LUT and Flip Flop (FF) and does not achieve the optimization effect. Block RAM, the custom block memory in the FPGA, does not consume combinatorial logic and sequential logic resources, which reduces resources consumption effectively. Therefore, we configure Block RAM as a dual-port Read-Only Memory (ROM), with a bit width of 8 bits and memory depth of 256 bits. The byte in the State is used as the input address and the data of the corresponding address in the S-box is used as the output. A dual-port ROM can perform two bytes SubBytes within a clock, while the single-port ROM can only perform one byte replacement with the same data bit width and memory depth; compared to single-port ROM, the dual-port ROM requires only half of the number of instantiations, effectively improving the utilization of Block RAM and reducing the memory resources consumption by half.

#### 3.2.2. MixColumns/Inverse MixColumns

MixColumns operates considering each column of the State. Based on the addition and multiplication of GF(2^8^), each column is treated as a polynomial. The data is multiplied by a given polynomial c(*x*) = 03 × ^3^ + 01 × ^2^ + 01*x* + 02 and then modulo the polynomial *x*^4^ + 1. The process can be represented by the matrix transformation Equation (1).

(1) $$\left[\begin{array}{c} s_{0}^{\prime }\\ s_{1}^{\prime }\\ s_{2}^{\prime }\\ s_{3}^{\prime } \end{array}]=[\begin{array}{cccc} 02 & 03 & 01 & 01\\ 01 & 02 & 03 & 01\\ 01 & 01 & 02 & 03\\ 03 & 01 & 01 & 02 \end{array}][\begin{array}{c} s_{0}\\ s_{1}\\ s_{2}\\ s_{3} \end{array}\right]$$

Similarly, the Inverse MixColumns of the decryption process changes the given polynomial c(*x*) to c^−1^(*x*) = 0B*x*^3^ + 0D*x*^2^ + 09*x* + 0E, which can be represented by the matrix transformation Equation (2).

(2) $$\left[\begin{array}{c} s_{0}^{\prime }\\ s_{1}^{\prime }\\ s_{2}^{\prime }\\ s_{3}^{\prime } \end{array}]=[\begin{array}{cccc} 0E & 0B & 0D & 09\\ 09 & 0E & 0B & 0D\\ 0D & 09 & 0E & 0B\\ 0B & 0D & 09 & 0E \end{array}][\begin{array}{c} s_{0}\\ s_{1}\\ s_{2}\\ s_{3} \end{array}\right]$$

In the GF(2^8^) finite field, the hardware implementation of multiplication is complicated, so we mainly optimize the operation. According to the principle of MixColumns, the constants in the coefficient matrix are 01, 02, and 03. Suppose *B*_0_ = (*b*_7_*b*_6_*b*_5_*b*_4_*b*_3_*b*_2_*b*_1_*b*_0_), then we can get {01} · *B*_0_ = *B*_0_, {03} · *B*_0_ = ({02} · *B*_0_⊕*B*_0_). It can be concluded that the multiplication of the finite field in MixColumns can be simplified as a multiplication of 2 and XOR operation, as shown in Equation (3).

(3) $$\left\{ 02\}\cdot \{b_{7}b_{6}b_{5}b_{4}b_{3}b_{2}b_{1}\}=\{\begin{array}{cc} \left\{b_{6}b_{5}b_{4}b_{3}b_{2}b_{1}0\right\} & b_{7}=0\\ \left\{b_{6}b_{5}b_{4}b_{3}b_{2}b_{1}0\}⊕\{00011011\right\} & b_{7}=1 \end{array}\right.$$

The FPGA implementation with multiplication by 2 operation can be realized by shift, XOR, and selection circuit, which is called “MixFunc_2(x)”. It is finally reduced to Equation (4). The four bytes input data are {*B*_0_, *B*_1_, *B*_2_, *B*_3_}, and the transformed data are {$B_{0}^{\prime },\;B_{1}^{\prime },\;B_{2}^{\prime },\;B_{3}^{\prime }$}.

(4) $$\begin{array}{l} B_{0}^{\prime }=\mathrm{MixFunc}\_2(B_{0})⊕(\mathrm{MixFunc}\_2(B_{1})⊕B_{1})⊕B_{2}⊕B_{3}\\ B_{1}^{\prime }=B_{0}⊕\mathrm{MixFunc}\_2(B_{1})⊕(\mathrm{MixFunc}\_2(B_{2})⊕B_{2})⊕B_{3}\\ B_{2}^{\prime }=B_{0}⊕B_{1}⊕\mathrm{MixFunc}\_2(B_{2})⊕(\mathrm{MixFunc}\_2(B_{3})⊕B_{3})\\ B_{3}^{\prime }=(\mathrm{MixFunc}\_2(B_{0})⊕B_{0})⊕B_{1}⊕B_{2}⊕\mathrm{MixFunc}\_2(B_{3}) \end{array}$$

In this paper, the optimized 3DES and AES-128 are implemented on ZYNQ-7000 series FPGA, respectively, recording the clock period constraint, clock period remains, and the Slice resources consumption, calculating the throughput and Throughput Per Slice (TPS), according to the Formulas (5) and (6). The results are shown in Table 2.

(5) $$\mathrm{Throughout}=\frac{\text{Date Block Length}\times \text{Clock Frequency}}{\text{Clock Cycles Per Encrypted or Decrypted Block}}$$

(6) $$\mathrm{TPS}=\frac{\text{Encryption or Decryption Rate}}{\text{CLB Slices Used}}$$

## 4. Dynamically Reconfigurable Encryption and Decryption System

In order to realize the dynamic switching between AES and 3DES, we have designed a Dynamic Reconfigurable Encryption and Decryption System. The whole system consists of an upper computer, reconfigurable control platform, and external memory. The Reconfiguration Control Platform supports the dynamic reconfiguration of cryptographic algorithms. Under the control of the Core Controller, the encryption algorithm is configured from the external memory to the Reconfigurable Partition. When the upper computer instructions have been received, the data encryption and decryption works are completed on the Reconfiguration Control Platform. This paper refines the execution function of each part of the proposed system and the specific dynamic reconfiguration process of the cryptographic algorithm.

### 4.1. Overall Structure

**Figure 3:**
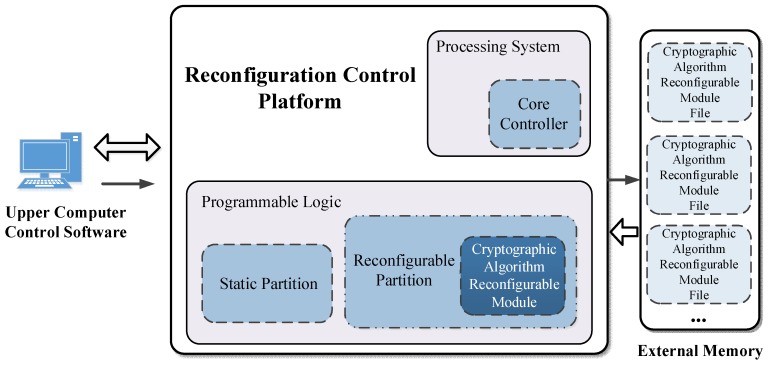
The overall structure of the DREDS.

As the overall structure of the DREDS is displayed in Figure 3, there are three major parts: Upper Computer Control Software, External Memory, and Reconfiguration Control Platform. The function of the Upper Computer Control Software is to send the reconfiguration control instructions, receive the returned reconfiguration state, and transmit and display the encrypted or decrypted information. The External Memory is responsible for storing cryptographic algorithm reconfigurable module files. When the reconfiguration instruction has been received, one of the cryptographic algorithm reconfigurable module files is transferred to the Reconfigurable Partition. The Reconfiguration Control Platform masters the core of dynamic reconfiguration processing, which is composed of the Programmable Logic with rich logic elements and the Processing System including the Core Controller. Based on the logic resources of Programmable Logic, the functions are designed for system clock management, data communication, and operating state control. The Core Controller is utilized to realize the reconfigurable module files dynamic read-in, the configuration tasks dynamic dispatching, and the internal data accessing, so that both the placement and routing and the Arithmetic/Logic Unit (ALU) are updated and the specified functions can be configured completely to the assigned Logical Extent of the Programmable Logic.

### 4.2. Reconfiguration Control Platform

Based on different functional characters, the logical extent is divided to two parts: Static Partition (SP) and Reconfigurable Partition (RP). Counter to the SP, performing the same operations abidingly and maintaining the same logic during the normal work, the task that RP performs is variable. Therefore, each part is arranged different work according to function requirements.

**Figure 4:**
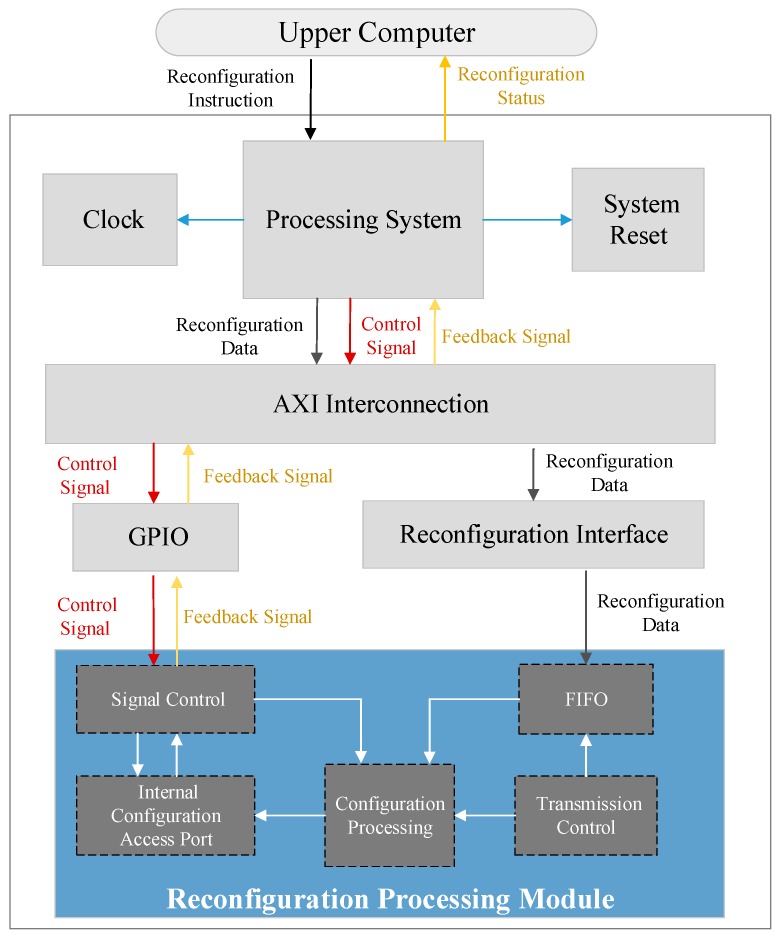
The architecture of the Static Partition (SP).

In the proposed system, because cryptographic algorithm reconfigurable module files could complete a dynamic swapping to download to Programmable Logic based on reconfiguration instruction, cryptographic algorithm reconfiguration logic is alterable, which should be part of the RP. The modules, including the processing system, the system reset, the clock module, the Advanced eXtensible Interface (AXI) interconnection, the General Purpose Input Output (GPIO), the reconfiguration interface, and the reconfiguration processing module execute tasks under the initial configuration, so they belong to the SP. The architecture of the SP is showed in Figure 4.

The processing system consists of application processing units, Double Data Rate SDRAM (DDR) controller, central interconnect, and multiple peripheral controllers, which is supported by Cortex-A9 processor. The assignment of the module is broken up into five steps:

1. The peripheral controllers is used for initializing peripherals and controlling working status.
2. The control instructions sent by the upper computer is received and the system status is returned to the upper computer through Universal Asynchronous Receiver/Transmitter (UART).
3. The control signal is generated to transmit to other modules according to the instructions.
4. The External Memory is controlled to transfer the selected cryptographic algorithm reconfigurable module file to DDR.
5. The reconfiguration control signal is generated, and transmitted the reconfiguration data to the RP.

The clock module and the system reset module generate clock and reset signal, respectively controlled by the processing system, according to the function demand of the system. The AXI interconnection is used to coordinate the different clock frequency between the processing system and the other modules, switch communication protocol and data-width, and cache transmitting data temporarily, ensuring the intermediate data reliable. The function of GPIO is to transfer the signal produced by the control program software to the RP and receive the feedback reconfiguration status, cooperating to complete the whole reconfiguration process. In the reconfiguration interface, the synchronization instruction, data-width, and desynchronization instruction are switched and the reconfiguration data is transferred to reconfiguration processing module to complete dynamic reconfiguration.

**Figure 5:**
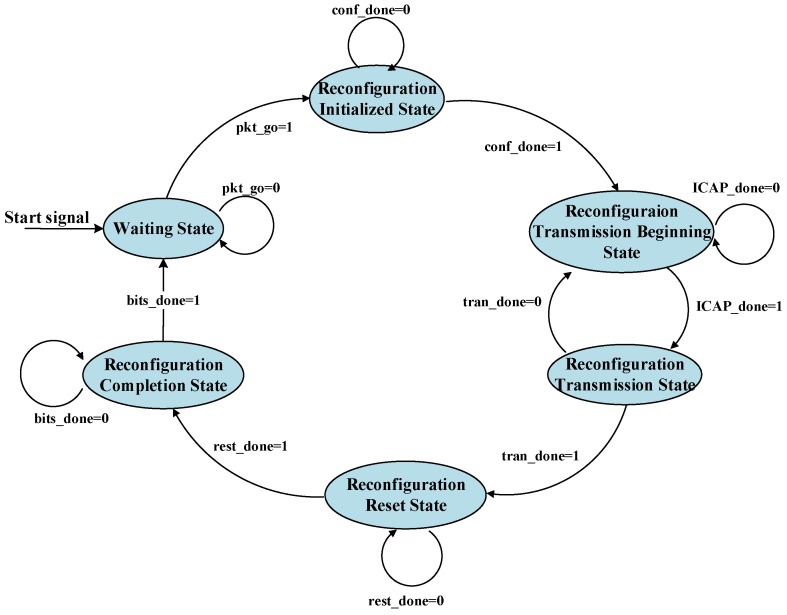
The state relation of the configuration state machine.

The reconfiguration processing module, the core of completing cryptographic algorithm reconfiguration to the RP, consists of five sections: The signal control module; First In First Out (FIFO); the transmission control module; the internal configuration access port; and the configuration processing module. FIFO solves the problem of the reconfiguration processing module clock frequency mismatching the transmission interface module by connecting connecting the two clocks separately and reading and writing the dedicated FIFO clock. The reconfiguration data in FIFO readings and transmissions are implemented using the transmission control module. The control signals transmitted with GPIO are received directly by the signal control module, which are not required to be cached in FIFO. In sequence, a series of signals as the reconfiguration commands are produced by the signal control module: the reconfiguration beginning level signal, the reconfiguration reset signal, the internal configuration access port enable signal, the read-write enable signal, and the configuration state machine enable signal. The configuration processing module that cooperates with signal control module commands and internal configuration access port aids in writing cryptographic algorithm reconfiguration module files. The configuration processing module consists of the configuration state machine, as shown in Figure 5. Each state is illustrated as follows:

1. **Reconfiguration Initialization State:** The initialization setting of the internal configuration access port is completed and the read-write enable control signal is reset as the original value.
2. **Reconfiguration Transmission Beginning State:** The internal configuration access port is activated to alter to writing mode, ready for receiving the reconfiguration data from DDR to write in the Reconfigurable Partition.
3. **Reconfiguration Transmission State:** The transmission signal stored in DDR takes effect to control the bitstream sequence of cryptographic algorithm reconfigurable module transmitted from the memory. The logic is decoupled to stabilize the signal between SP and RP, ensuring the bitstream sequence is written correctly into RP.
4. **Waiting State:** The internal configuration access port is keeping in the writing state, the bitstream sequence is downloaded steadily into the Reconfigurable Partition to configure the logic circuit. In the meantime, the condition of the internal configuration access port output is under check. After the command completion flag of the bitstream sequence downloaded is received, the Waiting State is exited.
5. **Reconfiguration Reset State:** The synchronization reset is enabled to reset the new logic elements in the Reconfiguration Partition, keeping in the known status.
6. **Reconfiguration Completion State:** The decoupled logic is released and the internal configuration access port and DDR is disabled. After the reconfiguration process is completed, the cryptographic algorithm reconfigurable module starts to work.

To guarantee the reliability of the transmitted encryption or decryption data, the two data communication modules are designed for transmitting reconfiguration instruction and encryption or decryption data. The reconfiguration instruction is processed to Cortex-A9 ARM processor by the upper computer and transmitted through the local UART, rather than the Programmable Logic. The encryption or decryption data is transmitted directly by the upper computer control software to the reconfigurable module, through the UART of the Programmable Logic. This implementation ensures the reconfiguration control and encryption or decryption data transmission not affect each other. In addition, the transmission speed could be improved by the means of transmitting the data directly from the Programmable Logic.

### 4.3. Core Controller

**Figure 6:**
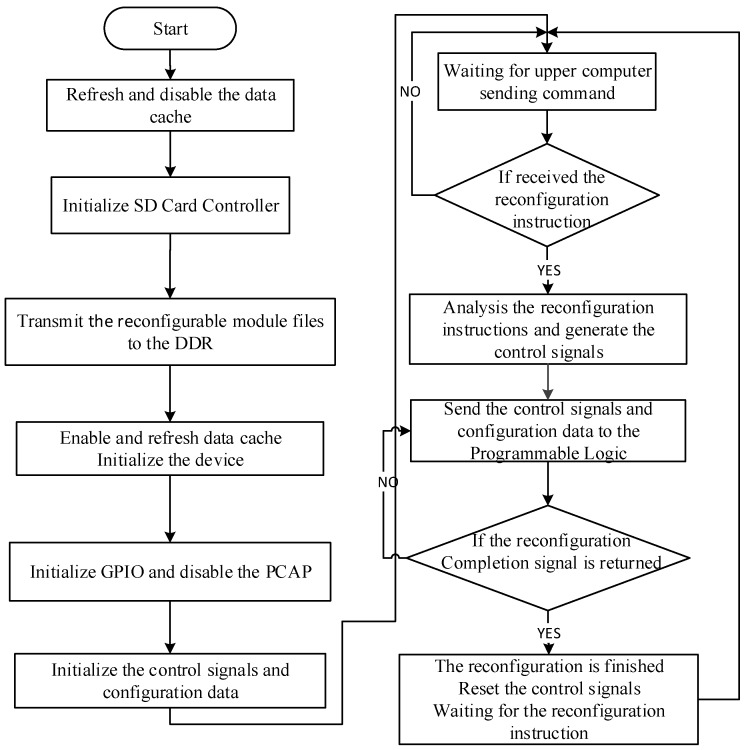
The program flow chart of Core Controller.

In the Cortex-A9 processor, the Core Controller as a control program software is designed to correctly configure the cryptographic algorithm reconfigurable module file from the External Memory to FPGA, in order to satisfy the requirements of the proposed system. The program flow chart of the Core Controller is displayed in Figure 6.

First, all the cryptographic algorithm reconfigurable module files stored in SD cards are transmitted using the SD card controller to the DDR through Direct Memory Access (DMA) under the software control. During the process, the SD card controller is initialized, to ensure the reconfiguration Bin files in the SD card could be identified correctly by the filename. Moreover, the address in the DDR should also be assigned, getting the utmost out of the memory space to match the reconfigurable module files. On account of the bus addresses manipulated directly by DMA, the data cache should be refreshed and disabled before transmitting data and enabled after transmission, in order to ensure the reconfigurable module files are entered correctly into the DDR to avoid writing back operation, which leads to data error between the cache and the DDR. The device is initialized after the data transmission is finished and the operation is performed as ID matching, base address setting, and device configuration interface opening, for ensuring the normal work. Next, the GPIO is initialized and the control signals and configuration data are assigned with initial values. Based on the design of writing reconfigurable module into Reconfigurable Partition through the Internal Configuration Access Port (ICAP), the Processing Configuration Access Port (PCAP) should be disabled to avoid data collision.

After the configuration has been completed, the control program software waits for the reconfiguration instructions sent by the upper computer. As the reconfiguration instruction is received, the instruction is analyzed and the signals corresponding to each module of Reconfiguration Control Platform are generated, then transmitted to the interconnection module through M_AXI_GP port. The reconfiguration process is controlled and the feedback state is monitored by the program. When the correct reconfiguration completion signal is returned, it indicates the reconfiguration process is finished. Then, the control signal is reset and it waits for the next reconfiguration instruction.

## 5. Results and Discussion

After the software simulation test passed, the hardware implementation of the DREDS is completed. The system’s encryption and decryption function and reconfigurable function are verified and the dynamically reconfigurable switching speed of the cryptographic algorithm using a logic analyzer is tested. In addition, we have conducted experiment on the SEDS and the DREDS to highlight the hardware resources consumption advantage of the proposed system.

### 5.1. Test Platform

**Figure 7:**
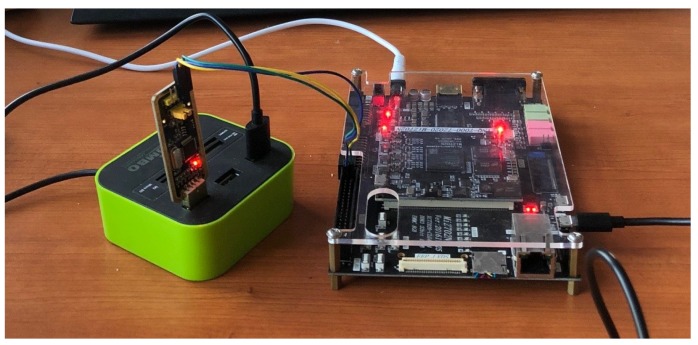
The experimental setup of the DREDS.

We implemented the proposed system on Xilinx’s ZYNQ-7000 series of FPGA, the model of xc7z020clg484-1. The series of ZYNQ-7000 is the extensible processing platform based on the Xilinx’s all Programmable SoC, integrating the Processing System including dual core Cortex-A9 ARM and the Programmable Logic with rich logical resources [29]. The dual core Cortex-A9 ARM as the core of the Processing System is composed of large quantities of memory and rich peripheral interfaces. The character of ZYNQ-7000 is that the specialized logic and the program could be mapped respectively to the Programmable Logic and the Processing System [30]. Based on requirements, the implementation between the hardware and the software could be balanced, in order to work cooperatively and achieve higher performance of the project designed. The experimental setup of our proposed system is displayed in Figure 7.

### 5.2. Data Validation

**Figure 8:**
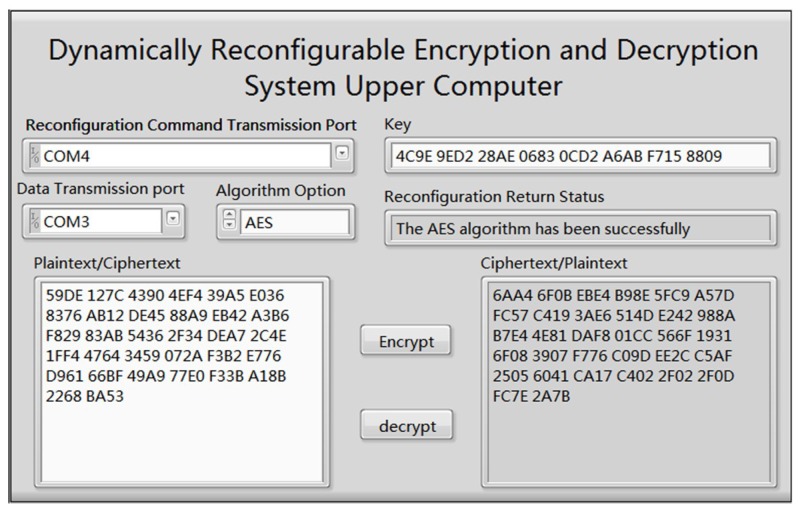
The interface of the Upper Computer Control Software.

We implemented the optimized AES and 3DES algorithms on the proposed system. The Upper Computer Control Software is programmed on LABVIEW 2015 for testing the cryptographic algorithm reconfiguration process and analyzing the relevant performance index. The reconfiguration command transmission port connects to USB to URAT and transmits the reconfiguration command to Cortex-A9 ARM. The data transmission port is connected with the Normal Expand Port (NEP) by USB to URAT and the plaintext or ciphertext is transmitted directly to the reconfigurable module. After the reconfiguration and encryption or decryption has finished, the returned data results are displayed in Figure 8.

The type of cryptographic algorithms could be reconfigured by selecting the dropdown menu in the Algorithm Option. Once an algorithm is selected by its users, the Upper Computer Control Software will send the reconfiguration command to FPGA through the Reconfiguration Command Transmission Port. After the cryptographic algorithm reconfigured, the Reconfiguration Return Status will be shown in the interface, and the Key and Plaintext/Ciphertext will be transmitted to FPGA through the Data Transmission Port, proceeding encryption or decryption process. When the data result is received, we compare the feedback with the correct data resulted by the standard encryption/decryption tool. If the data expresses no difference, it indicates that the system works correctly.

After a system functional test, the kernel control signal and the reconfiguration return state signal get connected to the logic analyzer using the extension pin of FPGA. The Bin file size of each cryptographic algorithm reconfigurable module is 776,156 Bytes. In addition, the cryptographic algorithm reconfiguration process is measured as the configuration time of 49.25 ms. Under calculated, the transmission rate of the whole reconfiguration process is 15,759.51 Bytes/ms.

### 5.3. Resources Consumption

**Figure 9:**
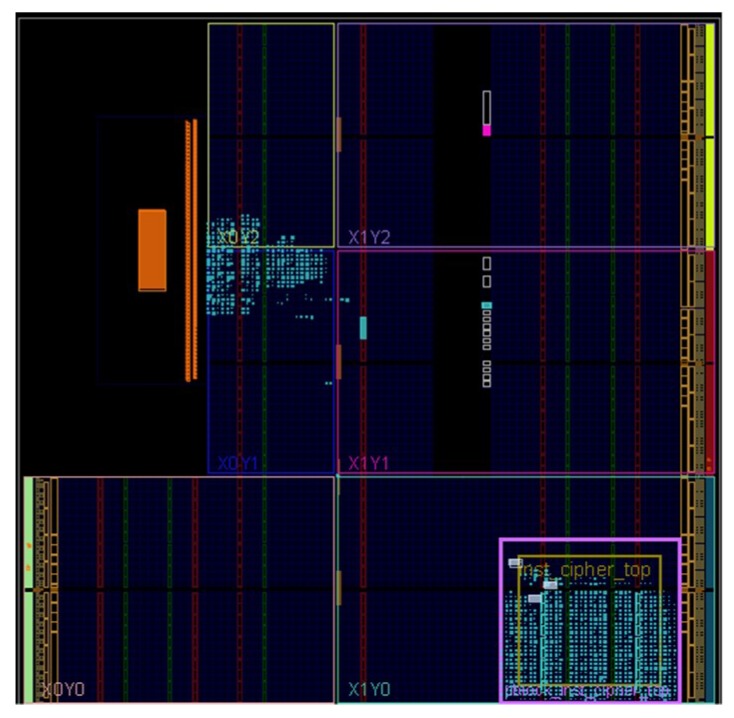
The resources occupancy of the proposed system.

Figure 9 shows the resources occupancy of the DREDS on xc7z020clg484-1, implemented on Vivado 2016.4. The blue lighted points in the figure is the used resources. The purple box is the cryptographic algorithm reconfiguration area, which is used to implement the AES or 3DES encryption and decryption. For convenience, we set AES as the default cryptographic algorithm. Table 3 records the resources usage number and the percentage of total resources of SP and RP under default algorithm implementation. It shows the use of the SLICE resources in the SP accounts for 2.42% of the total resources, which is only 39.31% of the RP resources consumption, indicating that the system still has large space to realize functions expansion. In other words, the entire system could complete encryption and decryption reconfiguration process with relatively few static configuration resources, which is of high practical value.

**Figure 10:**
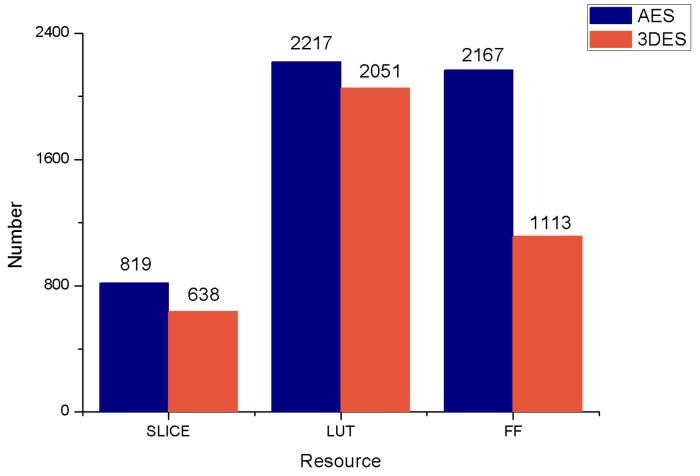
The resources utilization of each individual reconfigurable module.

The Reconfiguration Control Platform is the main part of the cryptographic algorithms switching. Resources reuse rate is one of the important technical indicators related to the performance of the entire system. For comparison, this paper analyzes the SLICE, LUT, and FF resource occupancy of the AES and the 3DES reconfigurable modules, respectively, before and after the reconfiguration. As shown in Figure 10, in the LUT resources consumption, the two module resources reuse rate is as high as 92.51%. The lower FF can also achieve 51.36% resources reuse rate, which indicates that the DREDS has high flexibility and good reconstruction performance.

### 5.4. Contrast with the SEDS

**Figure 11:**
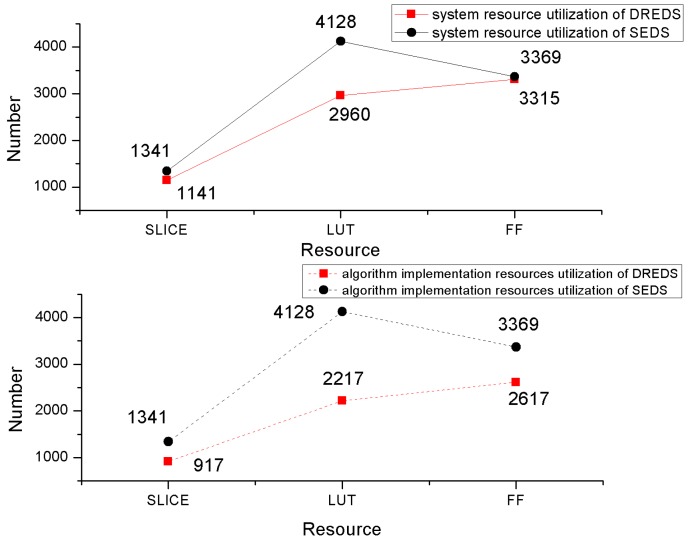
The system and algorithm implementation resource utilization of the two encryption and decryption systems.

In order to reflect the strengths of resources consumption and system flexibility in the proposed system, the detail data of DREDS and the SEDS is analyzed and contrasted. On the basis of [11], we have improved the SEDS implementation, not executing the encryption and decryption of 3DES and AES respectively, but the integration of AES and 3DES as one algorithm module, which reduces the hardware resources consumption. The algorithm selection control module is designed to select AES or 3DES to complete the encryption and decryption, according to the instructions of the upper computer. We have applied the two systems on the test platform and analyzed the resource utilization and algorithm implementation, respectively, using Vivado 2016.4 software. The corresponding data of each system is displayed in Figure 11.

The DREDS designed in this paper includes two parts: One, the reconstruction function supported by the SP and two, the algorithm reconfiguration realized by RP. However, the total system resource utilization of the two systems from Figure 11 show that our proposed system is still 14.91%, 46.29%, and 1.60% less than those in static design in SLICE, LUT, and FF resources. Combined with the SP and RP resources occupancy in Table 3, it can be understood that the proposed system takes all the logic optimization of the redundant algorithms in the SEDS except the selected algorithm as the SP to achieve the reconfiguration function, which greatly reduces the waste of resources.

The algorithm implementation resources utilization of DREDS has obvious advantages from Figure 11. It has 31.62%, 46.29%, and 35.68% less than the SEDS on SLICE, LUT, and FF logical resources, respectively. The reduced resources mainly come from the algorithm modules that are not used in SEDS. In the system designed in this paper, the modules are placed independently in the SD card, which is controlled by the upper computer software, saving a lot of space for FPGA limited on-chip resources.

## 6. Conclusions

In order to improve the information security and hardware processing performance of the IoT, we proposed multiple algorithms that support DREDS with high flexibility and high resource utilization, as well as the implementation of the Xilinx’s ZYNQ-7000 series of the FPGA. The Reconfiguration Control Platform, which was the most important part of the reconfiguration implementation, was designed with nine modules. The dynamic writing process of cryptographic algorithm reconfigurable module files was controlled by the configuration state machine. During the whole process, all modules worked cooperatively and implemented multiple cryptographic algorithms reconfiguration with the control of the Core Controller. In this paper, AES-128 and 3DES algorithm were implemented on the system and the Upper Computer Control Software was designed to control algorithm switching and data acquisition. Through data analysis and comparison with SEDS, our design not only realized the reduction of resources consumption on chip, but also ensured that the encryption and decryption system completed the switch of algorithm type at a high configuration speed in a stable working state. In addition, the system proposed in this paper supports more algorithms expansion into reconfigurable modules, aside from AES and 3DES. Considering that some IoT nodes do not require particularly complex application environments and ample resources, we will further research lightweight cryptographic algorithms into the DREDS and adapt the proposed system with the utilization of the Ethernet to realize long distance data communication.

## Figures and Tables

**Table 1:** The comparison between Dynamic Reconfigurable Encryption and Decryption System (DREDS) and the related works.

Works	High Flexibility	Multiple Cryptographic Algorithms Supporting	Cryptographic Algorithm Optimization	Low Resource Consumption
[6]	√ ^1^	×	√	×
[7]	× ^2^	×	√	√
[10]	√	√	×	×
[12]	×	√	×	√
DREDS	√	√	√	√

^1^ The feature is available in the work. ^2^ The feature is not available in the work.

**Table 2:** The logic resources consumption and data processing rate of the optimized 3DES algorithm and AES-128 algorithm.

Algorithms	Slice	Data Processing Rate			
		Clock Period Constraint	Clock Period Remain	Throughout	TPS
AES	689	5.000 ns	0.324 ns	2.74 Gbps	3.97 Mbps/Slice
3DES	412	5.000 ns	0.246 ns	280.47 Mbps	680.75 kbps/Slice

**Table 3:** The system resources consumption of the SP and RP in default situation.

Resources	Total Number	SP		PL	
		Number	Percentage	Number	Percentage
SLICE	13,300	322	2.42%	819	6.16%
LUT	53,200	743	1.40%	2217	4.17%
FF	106,400	1148	1.08%	2167	2.04%
BRAM	140	1	0.71%	9	6.43%
